# Evaluation of New Multimedia Formats for Cancer Communications

**DOI:** 10.2196/jmir.5.3.e16

**Published:** 2003-08-29

**Authors:** Judith L Bader, Nancy Strickman-Stein

**Affiliations:** ^1^National Cancer Institute, Communication Technologies BranchOffice of Communications, Cancer Information Products and ServicesBethesda MDUSA; ^2^Miami Veterans Affairs Medical Center (GRECC) and University School of MedicineDivision of Gerontology & Geriatric MedicineMiami FLUSA

**Keywords:** Lung cancer, Internet, multimedia, patient education, audio

## Abstract

**Background:**

Providing quality, current cancer information to cancer patients and their families is a key function of the National Cancer Institute (NCI) Web site. This information is now provided in predominantly-text format, but could be provided in formats using multimedia, including animation and sound. Since users have many choices about where to get their information, it is important to provide the information in a format that is helpful and that they prefer.

**Objective:**

To pilot and evaluate multimedia strategies for future cancer-information program formats for lay users, the National Cancer Institute created new multimedia versions of existing text programs. We sought to evaluate user performance and preference on these 3 new formats and on the 2 existing text formats.

**Methods:**

The National Cancer Institute's "What You Need to Know About Lung Cancer" program was the test vehicle. There were 5 testing sessions, 1 dedicated to each format. Each session lasted about 1 hour, with 9 participants per session and 45 users overall. Users were exposed to the assigned cancer program from beginning to end in 1 of 5 formats: text paperback booklet, paperback booklet formatted in HTML on the Web, spoken audio alone, spoken audio synchronized with a text Web page, and Flash multimedia (animation, spoken audio, and text). Immediately thereafter, the features and design of the 4 alternative formats were demonstrated in detail. A multiple-choice pre-test and post-test quiz on the cancer content was used to assess user learning (performance) before and after experiencing the assigned program. The quiz was administered using an Authorware software interface writing to an Access database. Users were asked to rank from 1 to 5 their preference for the 5 program formats, and provide structured and open-ended comments about usability of the 5 formats.

**Results:**

Significant improvement in scores from pre-test to post-test was seen for the total study population. Average scores for users in each of the 5 format groups improved significantly. Increments in improvement, however, were not statistically different between any of the format groups. Significant improvements in quiz scores were seen irrespective of age group or education level. Of the users, 71.1% ranked the Flash program first among the 5 formats, and 84.4% rated Flash as their first or second choice. Audio was the least-preferred format, ranking fifth among 46.7% of users and first among none. Flash was ranked first among users regardless of education level, age group, or format group to which the user was assigned.

**Conclusions:**

Under the pilot study conditions, users overwhelmingly preferred the Flash format to the other 4 formats. Learning occurred equally in all formats. Use of multimedia should be considered as communication strategies are developed for updating cancer content and attracting new users.

## Introduction

Seeking personal health information online is an increasingly-popular goal of Internet users [1,2], particularly cancer patients [3]. Providing critical but basic information in lay vocabulary to cancer patients and their families to help them make important personal health decisions is a key function of the National Cancer Institute's (NCI) Internet Web site [4]. Providing this information in the format users prefer and can learn from is also a priority, given the plethora of options and choices now available to consumers. To help develop and pilot strategies for developing content for this audience in the future, we sought to evaluate user experience with 5 different media formats of identical content. Three new media formats were created and evaluated as part of strategic decisions being made about how to offer content to the increasing number of users of broadband Internet connections.

Our hypotheses were that compared to users of the 2 existing, traditional, predominantly-text formats, users of newer media formats would (1) demonstrate more "learning" of complex cancer information and (2) prefer the learning experience.

This report describes (1) the initial pilot project creating 3 new media formats from previously-existing predominantly-text content, and (2) formal comparison of user learning (performance) and preference for the 3 new and 2 existing program formats.

The 5 media formats evaluated for this study were:

1. Paper (existing: paperback booklet, predominantly text)
2. Web (existing: paperback booklet in HTML format on the Web)
3. Audio (new: spoken audio files available for streaming or download)
4. Audio plus Web (new: spoken audio synchronized with existing Web page)
5. Flash (new: animation loops, graphics, synchronized sound, dictionary).

## Methods

As the vehicle for format comparisons, we selected NCI's booklet "What You Need to Know About Lung Cancer" [5], which is part of the "What You Need to Know About Cancer" program series [6], authored by NCI's Cancer Information Service [7]. This 26-booklet series provides basic information about cancer in general and information about 25 specific cancer sites information (causes, statistics, diagnosis, testing, treatment, outcomes, follow-up, clinical trials). It is targeted for readers with an 8th-grade to 10th-grade education. The series, originally published as mostly text in paperback booklet format, has recently been offered online in HTML format, duplicating the design and content of the paperbacks.

The "What You Need to Know About Lung Cancer" program was selected for this pilot for several reasons:

1. The annual incidence of new lung cancers is high [8].
2. The NCI lung cancer booklet seemed especially suitable for multimedia [6].
3. Other common cancers like breast [9] and prostate [10] already had many prominent portals on other Web sites.
4. NCI lung cancer content was not scheduled for rewrite for 2 years.
5. Other major patient-oriented online lung cancer Web sites [11- 13], including our own [5], do not take full advantage of multimedia features, even if multimedia software is used [14].
6. Recent data on newly-diagnosed lung cancer patients of all education and social strata demonstrated that they frequently search the Web to get information about their diagnosis [15].

Flash [16] software was selected as the format to create a program with animation loops, spoken audio, and text because about 92% of US computers have the Flash Player plug-in already installed [17]. NCI contracted with Medicom Digital, Inc [18] to create the Flash program, in collaboration with NCI content experts [19]. The joint team used existing program text (word for word) but created a new user interface, selected various features to include in the program, tested the program interface in formal usability tests, and rebuilt the interface based on testing results.

Audible, Inc was selected to create and host the newly-created spoken-audio files of the existing lung program, as well as other programs in the series [20]. The audio files recorded the existing program text word for word. Audio navigation links were added to offer users the option to jump to specific sections or listen from beginning to end. Users could listen by streaming or downloading content to a desktop computer or a personal digital assistant (PDA). For this study, files downloaded to the desktop computer were used.

The synchronized audio plus Web version combined the existing Web page and the new audio files.

The Web version of the text program [5] and the paperback booklet (available free by mail) [7] are both available through the NCI Web site [4].

A demonstration of the "look and feel" of each of the 5 formats is in Multimedia Appendix 1.

To test and compare how well prototypical users "learned" cancer content from each of the 5 formats, the development team used Authorware [21] software to create and present a 16-item multiple-choice test [Appendixes 2,3]. NCI staff prepared the quiz questions and answers based on the content in the text program. The Authorware interface also elicited and recorded demographic information, recorded and graded quiz answers, recorded time on each question, and recorded usability test information. All data were written to a Microsoft Access database.

To evaluate the individual programs before formal testing, an experienced facilitator, using a formal script, tested several users on each of the 5 program formats. Users were tested one at a time for an hour each, to evaluate usability and effect of the media programs and the quiz instruments, including the Authorware modules. Users of various ages, education levels, and Internet experience were included. These sessions found that almost all users showed learning from pre-test to post-test. This preliminary testing confirmed that participants were able to use the media-program software itself and the test software. Although this was not formal instrument validation, it does suggest that our data are reliable.

For final testing, there were 5 user testing sessions, with each session featuring 1 of the 5 format types. There were 9 users per session, and each session lasted about 1 hour. The procedure was the same for each session. In each of the 5 sessions, all 9 users took the Authorware pre-test on the cancer content. All 9 were then required to experience the entire lung cancer program from beginning to end in only 1 of the 5 formats — paper, Web, audio files, Web plus audio, or Flash. (In the testing version of the Flash program, the quiz questions were disabled.) After experiencing the entire program in that format, all 9 users viewed/heard a detailed demonstration of the key features and the look and feel of the other 4 formats. Then, each of the 9 users was asked to provide their answer to the following question: "If you needed to learn this lung cancer content for yourself and could get access to only 1 format, list in order from 1 to 5 your personal choices and tell us why you picked this order." Then users took the 16-item post-test quiz on the content. The quiz question order was the same between pre-test and post-test, but the order in which the answers were displayed was changed between the pre-test and post-test. At the conclusion of the session, users supplied additional usability data based on the primary format they experienced in the session. These data will be reported elsewhere.

As is typical for usability testing, 45 paid volunteers were selected by a nongovernmental market-research recruiting firm, based on a screening document supplied by the NCI research team. Balance among the groups for relevant parameters was requested. No recruit could have experienced cancer personally, had a close relative with lung cancer, or worked in medical science professions. English fluency was required. Balance of age, gender, formal education, and Internet computer experience among the groups was requested. The recruiting firm found and assigned all volunteer users to 1 of 5 groups, not knowing what media format they would be testing. The testing order of the media format groups was decided in advance by the research team without knowledge of who had been recruited for the groups. Strict randomization of users was not performed, but the search firm's recruits were generally balanced for the parameters requested by the research team (Table 1). Users signed the standard NCI consent to participate in usability testing of Web sites. To comply with US Office of Management and Budget restrictions on federal surveys, only 9 users could be recruited for each of the 5 program formats tested. Testing took place at NCI's new Communication Technologies Research Center, where each user had his/her own computer.

**Table 1:** Demographic characteristics of users by format to which each was assigned

**Assigned MediaFormat**	**Number of Users**	**Number of Users in Each Age group**	**Average Age**	**Age Range**	**Male:Female**	**Highest Education Level**
		**41-54**	**55-64**	**65-77**				**High School**	**Some College**	**College Graduate**	**Post College**
Paper	9	4	3	2	57.1	41-74	4:5	1	3	4	1
Web page	9	4	3	2	56.2	45-69	4:5	0	5	2	2
Audio	9	1	3	5	63.4	48-76	4:5	1	3	3	2
Audio plus Web	9	3	4	2	57.3	42-77	5:4	3	2	1	3
Flash	9	2	5	2	58.6	47-65	5:4	1	3	1	4
Total or Range	45	14	18	13	58.5	41-77	22:23	6	16	11	12

### Statistical Analyses

Paired *t*-tests and ANOVA were performed to assess the relationships between pre-tests and post-tests of content knowledge among the users of each media format. One-way ANOVA and Fisher's Least Significant Difference (LSD) post-hoc tests were performed to assess differences in performance from pre-test quiz scores to post-test quiz scores between the different media-format groups. Chi-square tests were performed to assess the association between media preference both by age group and by education level.

## Results

### Demographics of Study Participants

Table 1 summarizes the demographic characteristics of the 45 users according to the format to which they were assigned for the main presentation. Mean age was 58. Gender was equally distributed among the groups. Only 6 of 45 users had a highest-education level of high school and 12 had post-college education. Characteristics among the 5 groups reflect the demographics of Montgomery County, Maryland, where the testing occurred.

### Quiz Scores (Performance)

To assess how well users learned the cancer content presented by the media format to which they were assigned, a 16-item multiple-choice Authorware pre-test and post-test quiz was administered to each user. Sixteen was a perfect score. No differences in pre-test scores were seen between groups at baseline. Table 2 summarizes the pre-test and post-test scores for each of the 5 groups to which users were assigned, and for the group as a whole. Significant improvement was seen within each group. Only 4 users did not improve their scores: a 47-year-old high school graduate assigned to paper, a 74-year-old with some college education assigned to audio, a 56-year-old with a college education assigned to audio, and a 77-year-old with post-college education assigned to audio plus Web. No trend is apparent based on these 4 users.

**Table 2:** Quiz scores tabulated by assigned media format group*

**Assigned media format**	**MeanPre-test Score**	**MeanPost-test Score**	***t*(df)**	***P***
Paper	7.78	10.56	3.49 (8)	<< .01
Web	6.89	10.00	3.97 (8)	<< .01
Audio	7.67	10.67	3.34 (8)	.01
Audio plus Web	8.00	11.40	5.00 (8)	<< .01
Flash	7.60	11.80	4.18 (8)	<< .01
Total	7.44	10.91	8.72 (44)	<< .01

^*^ An analysis of variance indicated no differences in improvements between the different groups (F _4,40_= 0.598, *P*=.67).

### Quiz Scores Among Special User Groups

Because of NCI's special interest in older users and those with less formal education, quiz scores for these groups were analyzed separately. Thirteen participants were age ≥65. The mean pre-quiz score for this group was 7.23, while the mean post-test score was 10.62. This represents a significant improvement ( *t*_12_= 6.03, *P*< .001). Significant improvements in pre-test to post-test scores were seen for each of the age groups (41-54, 55-64, ≥65). However, the increments in improvement were not significantly different between any of the 3 age groups (F _2,42_= 0.266, *P*= .77). There were too few study participants to compare statistical improvement in scores by age group and assigned format.

Six participants had a high school education. The mean pre-test score for this group was 6.50, while the mean post-test score was 10.50. This represents a significant improvement ( *t*_5_= 3.38, *P*= .02). Significant improvements in pre-test to post-test scores were seen for each of the 4 education levels defined in Table 1. However, the increments in improvement were not significantly different between any of the 4 education levels (F _3,41_= 0.872, *P*= .47). There were too few study participants to compare statistical improvement in scores by education level and assigned format.

### User Format Preferences

Each of 45 users was asked to provide a ranking from 1 to 5 of the format they preferred for the lung cancer program. Preference data are shown in Table 3. Participants overwhelmingly preferred the Flash format. Thirty-two of 45 users (71.1%) selected Flash as their first choice, and 38 of 45 (84.4%) rated Flash as either their first or second choice. Five individuals selected Flash as their fifth choice (11.1%). Audio was the least preferred format, ranking 5th among 21 of 45 (46.7%) users. Audio was not the first or second choice for any participant.

**Table 3:** Format choices for each of 45 users

**Format Choices**	**Number of Users Selecting Each Ranking**				
****	1st	2nd	3rd	4th	5th
Flash	32	6	1	1	5
Paper	4	12	9	10	10
Web	4	8	17	13	3
Audio plus Web	5	19	10	5	6
Audio	0	0	8	16	21

User choices were also evaluated by the format to which users were assigned (Table 4). Flash was selected first by 8 of 9 users in the Flash group, 6 of 9 users in the Web group, 6 of 9 users in the paper group, 4 of 9 users in the audio group, and 8 of 9 users in the audio plus Web group.

**Table 4:** User choices of media by assigned format

**9 Multimedia Users Choices**						
**Choice**	**1st**	**2nd**	**3rd**	**4th**	**5th**	
Flash	8	0	0	1	0	
Paper	0	4	0	2	3	
Web	1	1	5	2	0	
Audio plus Web	0	4	2	1	2	
Audio	0	0	2	3	4	
**9 Paper Users Choices**						
**Choice**	**1st**	**2nd**	**3rd**	**4th**	**5th**	
Flash	6	2	0	0	1	
Paper	1	0	4	1	3	
Web	1	3	1	4	0	
Audio plus Web	1	4	3	1	0	
Audio	0	0	1	3	5	
**9 Web Users Choices**						
**Choice**	**1st**	**2nd**	**3rd**	**4th**	**5th**	
Flash	6	3	0	0	0	
Paper	1	1	3	2	2	
Web	2	2	3	2	0	
Audio plus Web	0	3	2	2	2	
Audio	0	0	1	3	5	
**9 Audio plus Web Users Choices**						
**Choice**	**1st**	**2nd**	**3rd**	**4th**	**5th**	
Flash	8	0	0	0	1	
Paper	0	4	1	3	1	
Web	0	1	4	2	2	
Audio plus Web	1	4	3	1	0	
Audio	0	0	1	3	5	
**9 Audio Users Choices**						
**Choice**	**1st**	**2nd**	**3rd**	**4th**	**5th**	
Flash	4	1	1	0	3	
Paper	2	3	1	2	1	
Web	0	1	4	3	1	
Audio plus Web	3	4	0	0	2	
Audio	0	0	3	4	2	
**Totals for All 45 Users**						
**Choice**	**1st**	**2nd**	**3rd**	**4th**	**5th**	**Totals**
Flash	32	6	1	1	5	45
Paper	4	12	9	10	10	45
Web	4	8	17	13	3	45
Audio plus Web	5	19	10	5	6	45
Audio	0	0	8	16	21	45
Totals:	45	45	45	45	45	

Open-ended comments by users typically indicated they liked the Flash format because of its rich visual content, because they considered themselves "visual" learners, and because they thought it would be easiest to learn the complex content when animations, pictures, and sound were used instead of just text. Users who liked the paperback format typically noted its portability and independence from the computer. Users who liked the Web version said that they liked this version because they were familiar with how Web pages worked and knew how to print them out. Users who liked the audio plus Web format said they thought it helped them learn by reading and having the material read to them at the same time. Users said they "disliked" the audio alone format generally because it was hard to remain attentive for the entire program from beginning to end as required by the study methodology, and they found it difficult to navigate among program sections. Others suggested that downloaded audio might be useful while traveling, in a car or other vehicle, where Internet connections are not available. Users also commented that their elderly relatives might have preferred the audio format, because it was "like radio," something with which they were very familiar and comfortable. Regardless of the format group users were exposed to, they liked the content, felt they learned from it, and appreciated that it was made available to them by the NCI.

Because of NCI's special interest in older users and those with less education, preference data for these groups were analyzed separately.

### Format Preference by Age Group

All age groups reliably selected Flash as their first choice of media format. Table 5 demonstrates that participants from the youngest, middle, and oldest age categories overwhelmingly preferred the Flash format. Although a higher proportion of participants ages 55 to 64 selected Flash as their first choice for format, this proportion was not significantly different than reported by those in the other age group categories (Χ ^2^_6_= 8.32, *P*= .216). No users picked audio as their first or second choice.

**Table 5:** First choice of format by age group

**Age Group**	**First Choice of Format**			
	**PaperNo. (%)**	**WebNo. (%)**	**Audio plus WebNo. (%)**	**FlashNo. (%)**
41-54	0 (0.0)	2 (14.3)	3 (21.4)	9 (64.3)
55-64	1 (5.6)	2 (11.1)	1 (5.6)	14 (77.7)
65+	3 (23.1)	0 (0.0)	1 (7.7)	9 (69.2)
Total	4 (8.9)	4 (8.9)	5 (11.1)	32 (71.1)

### Format Preference by Education Level

Table 6 illustrates first choice of formats by education level. Participants with a high school education level tended to prefer the Flash format. Four of 6 (66.7%) chose Flash as their first choice. Five of the 6 (83.3%) chose Flash as either their first or second choice of format. These data illustrate that the participants preferred the Flash format regardless of their personal education level. No significant differences in format preference were seen between the different education levels (χ ^2^_9_= 8.32, *P*= .216). No users selected audio as their first or second choice.

**Table 6:** First choice of format by education levels

**EducationLevel**	**First Choice of Format**			
	**PaperNo. (%)**	**WebNo. (%)**	**Audio plus WebNo. (%)**	**FlashNo. (%)**
High school	0 (0.0)	0 (0.0)	2 (33.3)	4 (66.7)
Some college	1 (6.3)	1 (6.3)	2 (12.5)	12 (75.0)
College	2 (18.2)	1 (9.1)	1 (9.1)	7 (63.6)
Post college	1 (8.3)	2 (16.7)	0 (0.0)	9(75.0)
Total	4 (8.9)	4 (8.9)	5 (11.1)	32 (71.1)

## Discussion

This study evaluated user performance and preference on 5 formats of identical NCI lung cancer content. The most-significant findings were that (1) users in every format group improved their test scores significantly and (2) users overwhelmingly preferred the Flash format for this content. These findings were true regardless of age or education level. The pre-test and post-test quiz score data suggest that the content was useful and valuable, which corresponds with users' open-ended comments.

We had hoped to find, but did not find, a significant improvement in learning (quiz performance) with Flash users compared to other formats. There are several possible reasons:

1. There was no significant difference in learning due to media format.
2. The Flash format we created did not optimize the teaching potential of that format.
3. There were too few users overall, or too few users with specific learning styles to detect small but significant learning differences favoring Flash.
4. The multiple choice quiz used was an inadequate instrument to detect real learning differences among the formats tested.
5. Requiring users to experience the lung cancer program from beginning to end did not replicate normal user learning behavior with any of the various formats.
6. Test participants may not have reflected learning that would have occurred among a different and more highly-motivated group of actual cancer patients and their families.

Summarizing considerable research on multimedia and e-learning, Mayer has suggested that "questions about which medium is best (for teaching) are somewhat unproductive." He states that "in general, media effects are small . . . it is not possible to separate the effects of the medium from the effects of instructional method . . . learning outcomes depend on the *quality of the instructional method rather than on the medium per se*(emphasis added)" [22]. Dillon and Babbard, in an extensive review of educational research, indicate that the benefits of hypermedia learning are "differently distributed across learners depending on their ability and preferred learning style" [23]. Najjar's review of multimedia and learning suggests that multimedia information is most effective when "presented to learners with low prior knowledge or aptitude in the domain being learned" [24]. Most newly-diagnosed cancer patients fit this profile. Mayer's data also confirm differential effects of specific multimedia formats on learners with specific learning styles [25].

Complex health information can be very difficult to convey to patients newly diagnosed with serious illnesses, such as cancer. The message may be difficult to transmit in a meaningful way, individuals do not always want to receive the message, and anxiety may interfere with learning. The strong preference data supporting Flash suggest that information seekers may be more receptive to a cancer message using this format, which would potentially have an advantage in attracting and keeping user interest.

Our study data confirm other findings that older adults are receptive to learning through multimedia formats [26]. On the other hand, our data also show that learning is independent of format. Therefore, offering the same content in audio alone or audio plus Web, although less popular in our study, might still be preferred by a large number of users, given the absolute number of newly-diagnosed cancer patients annually who seek basic information.

Research has also shown that cancer patients often desire more information than they receive and that the format in which they receive the information should be based on their preference [27].

The fact that no differences were detected in either quiz performance or format preference by personal education levels further emphasizes the potential global appeal of the Flash multimedia approach. This is consistent with other findings that education level does not predict reading ability, and that the desire for information is the critical component [28].

Over 150 Flash multimedia tutorials on many health topics [29], including lung cancer [14], are available online from MEDLINEplus, a service of the National Library of Medicine (NLM) [30]. These tutorials are smaller files, and may be accessible with smaller bandwidth than ours, but they do not provide as much content depth as our pilot [19]. We are unaware of any data published by NLM about effectiveness of these tutorials as teaching instruments, although internal data have suggested that they are very popular (:Elliot Siegel, PhD; NLM; oral communication, 2003).

Having validated ease of program use during the development of the Flash program interface and preference for Flash during this study, we suggest that there is value in continuing to use and improve the interface. User testing revealed appreciation of specific program features including animation loops, spoken dictionary, selective printing of graphics and chapters, internal quizzes for review, chapter outlines, and a full audio text in addition to the graphic features.

Considering users' strong preference for the new Flash program, we can envision other uses for the Flash interface in cancer education such as augmenting pure-text informed consents, teaching about clinical trials, explaining medical procedures, teaching about healthy behaviors, engaging children in content learning, and non-English language presentations. The interface could also be helpful to groups other than the general public. Currently, complex NCI content for genetics professionals is being programmed using our Flash interface.

We hope to continue to test additional multimedia prototypes among various user groups, including those with accessibility issues. In the future, we hope to perform usability testing on low-vision users to assess their reaction to the new spoken-audio files, which we suspect may be more pleasant to listen to than a synthesized screen reader. We also hope to test the Flash program with low-hearing users because it has a complete and synchronized audio-text option available as users watch the animations. Federal regulations require compliance with accessibility regulations. Offering the programs in multiple formats ensures that we remain compliant.

One potential problem with the current Flash program is its large file size, making it available only to those with a broadband Internet connection. For this reason, consideration is being given to making it available on CD. According to recent data, wide bandwidth is available at home to 17% to 28% of users and the number is increasing [17,31]. Users in the workplace, including those in medical offices and hospital cancer-resource centers for patients, probably have access to higher bandwidth [17,31]. Users clearly need and search for cancer information online [1,2]. As more users acquire access to the Internet via wide-bandwidth connections, it becomes increasingly important to provide the content users want in the format they prefer, especially given the wide number of choices of cancer content online. We are aware of excellent commercially-produced anatomical site-based cancer multimedia programs using sound, animation, and film clips [32]. At present, the file sizes are so large that the programs are available only on CD, and their content is targeted at a much-higher reading level than ours.

Accessibility of multimedia programs is an issue with respect to Section 508 guidelines for US government Web sites [33] and compliance with http://www.w3.org/WAI/(WAI) guidelines [34]. Complex multimedia offerings like ours, if offered in isolation, could fail to comply with the published guidelines. It is our hope to offer multiple links to the same content in different media formats on the same Web page. With compliant programming techniques and proper link labels both to and within the multiple media program options for the lung cancer content on the appropriate cancer.gov Web page, we expect that those with visual, auditory, or motor disabilities could choose the format that works best for them, and the spirit of compliance would be fulfilled. Additional testing of the multimedia formats with various disabled user groups is planned.

Our study could be faulted for its small numbers. From the outset, we intended the project as a small pilot study. In addition, the US Office of Management and Budget (OMB) regulations restrict any survey of citizens to ≤9 users per project without a special OMB waiver which is generally difficult and time consuming to obtain for a study of this type. Furthermore, the content would potentially need to change before the waiver was obtained. To comply with these restrictions, we were allowed to survey only 9 users for each format. Recruiting users online would have been the most-efficient and cheapest way to recruit large numbers of users. Even with an OMB waiver, we did not think it would be feasible to ask Internet users, even compensated, to compare online by themselves all 5 formats of the identical program. Most importantly, it was the format comparison data which was of special interest in the planning for future NCI communication products. Although we would have liked to survey additional users with less formal education and older age, the data gathered did suggest very-specific user preferences and significant learning with both new and older media formats. Nonetheless, the study was able to evaluate improvements in knowledge and performance for each of the 5 media formats, 4 education levels, and 3 age groups. The sample size was not large enough, however, to detect statistically-significant differences in improvements between any of the media groups, either alone or by subgroup stratum. Relatively-uniform increases in improvements were seen among all participants, and therefore the detected differences in our study were too small to be considered educationally important. Therefore, the benefit from increasing the sample size would not have resulted in improved overall results. However, an increased sample size would have allowed for the analysis of media formats by the different strata of age and education.

In conclusion, evaluation of 5 formats of identical NCI lung cancer content targeted for the general public in this pilot study suggested that users learned well with all 5 formats but preferred the new Flash multimedia tutorial format overwhelmingly. Multimedia content using animation and sound need not be created in Flash, but it should take advantage of sound and useful graphics and animation loops to communicate effectively and interestingly with users. Embedding Flash movies or other multimedia animation loops inside text Web pages might also provide learning assistance without having to create entirely-new stand-alone programs, and the components may be reusable in many programs. Given the large number of newly-diagnosed cancer patients annually, providing choices of media formats would allow learners of many different styles to maximize their chance of learning the information they need. By providing valuable content and maintaining user interest, new media options show promise in fulfilling the NCI mission of educating citizens about what they need to know about cancer in the format they prefer.

## Abbreviations

NCI: National Cancer Institute
NLM: National Library of Medicine
OMB: Office of Management and Budget

## Multimedia Appendix 2

### Content quiz questions andanswers (correct answer is indicated with an asterisk)

Quiz Questions

Question 1:

Benign lung tumors:

- Usually require treatment with chemotherapy
- Spread slowly to distant organs
- Grow through a process called metastasis
- Can often be removed *

Question 2:

Small cell lung cancer:

- Usually grows more slowly than non-small cell cancers
- Is also called oat cell cancer *
- Is less likely to spread to other organs than non-small cellcancer
- Is more common than non-small cell cancers

Question 3:

Each of the following increases the chance ofgetting lung cancer EXCEPT:

- Exposure to radon
- Smoking pipes
- Exposure to people who have lung cancer *
- Exposure to asbestos

Question 4:

All of the following are common symptoms of lungcancer EXCEPT:

- Difficulty swallowing *
- Weight loss
- Persistent cough
- Chest pain

Question 5:

A biopsy for lung cancer usually involves thefollowing:

- Lung scan
- Removal of tissue from the lung *
- Microscopic examination of sputum
- Internal radiation

Question 6:

All of the following are useful diagnostic tests forlung cancer EXCEPT:

- Needle aspiration
- Bronchoscopy
- Thoracentesis
- Internal radiation *

Question 7:

Lung cancer staging is done to:

- Determine if and where the cancer has spread *
- Decide which diagnostic tests to perform
- Evaluate the biopsy report
- Determine if the cancer will respond to treatment

Question 8:

All of the following are commonly used imaging testsfor lung cancer EXCEPT:

- MRI
- CAT scan
- External radiation *
- Bone scan

Question 9:

Mediastinoscopy and mediastinotomy are proceduresthat:

- Remove a sample of the fluid that surrounds the lungs tocheck for cancer cells
- Help show whether cancer has spread to the lymph nodes in thechest *
- Remove a portion of the tissue inside the lung
- Insert a needle into the tumor in the chest to remove asample of lung tissue

Question 10:

All of the following are types of surgery used totreat lung cancer EXCEPT:

- Wedge resection
- Lobectomy
- Segmental resection
- Mediastinotomy *

Question 11:

Chemotherapy for patients with lung cancer:

- Is most effective when injected directly into the lung
- Has very limited side effects
- Affects both normal and cancer cells *
- Is only administered into a vein

Question 12:

All of the following are true about radiationtherapy for patients with lung cancer EXCEPT:

- Affects cancer cells inside and outside the treated area*
- Can be given internally
- May be used before or after lung surgery
- Can be given with other kinds of treatments for lungcancer

Question 13:

All of the following are commonly caused bytreatment for lung cancer EXCEPT:

- Mouth sores
- Nausea and vomiting
- Weight gain *
- Fatigue

Question 14:

All of the following accurately describe clinicaltrials for lung cancer EXCEPT:

- Locate lung cancer clinics *
- Are described on the National Cancer Institute's website
- Can compare a new therapy to a standard therapy
- Are appropriate for patients with non-small cell lungcancer

Question 15:

All of the following are common treatments for smallcell lung cancer EXCEPT:

- Chemotherapy
- Radiation therapy to the lung
- Radiation therapy to the brain
- Surgery *

Question 16:

All of the following are common treatments fornon-small cell lung cancer EXCEPT:

- Bronchoscopy *
- Thoracentesis
- Radiation therapy
- Chemotherapy
