# Supplementary material for: Evaluation of New Multimedia Formats for Cancer Communications
Source: J Med Internet Res. 2003 Aug 29;5(3):e16. doi: 10.2196/jmir.5.3.e16 (PMC1550563; doi:10.2196/jmir.5.3.e16)
Supplement: Supplementary file 1 [file jmir_v5i3e16_app1.ppt]

## Slide 1
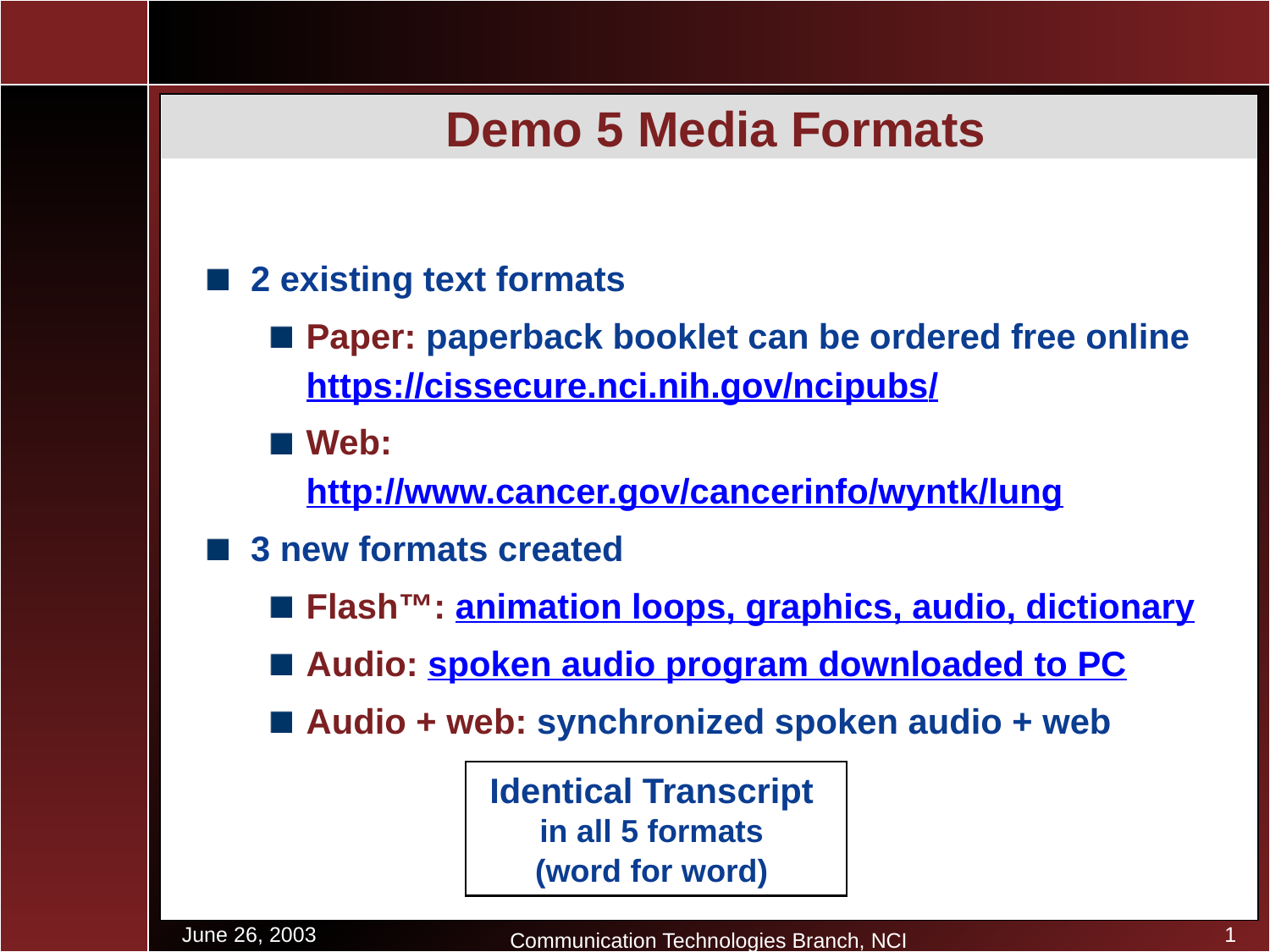

# Demo 5 Media Formats
2 existing text formats
Paper: paperback booklet can be ordered free online https://cissecure.nci.nih.gov/ncipubs/
Web: http://www.cancer.gov/cancerinfo/wyntk/lung
3 new formats created
Flash™: animation loops, graphics, audio, dictionary
Audio: spoken audio program downloaded to PC
Audio + web: synchronized spoken audio + web
Identical Transcript
in all 5 formats
(word for word)
June 26, 2003
1
Communication Technologies Branch, NCI

## Slide 2
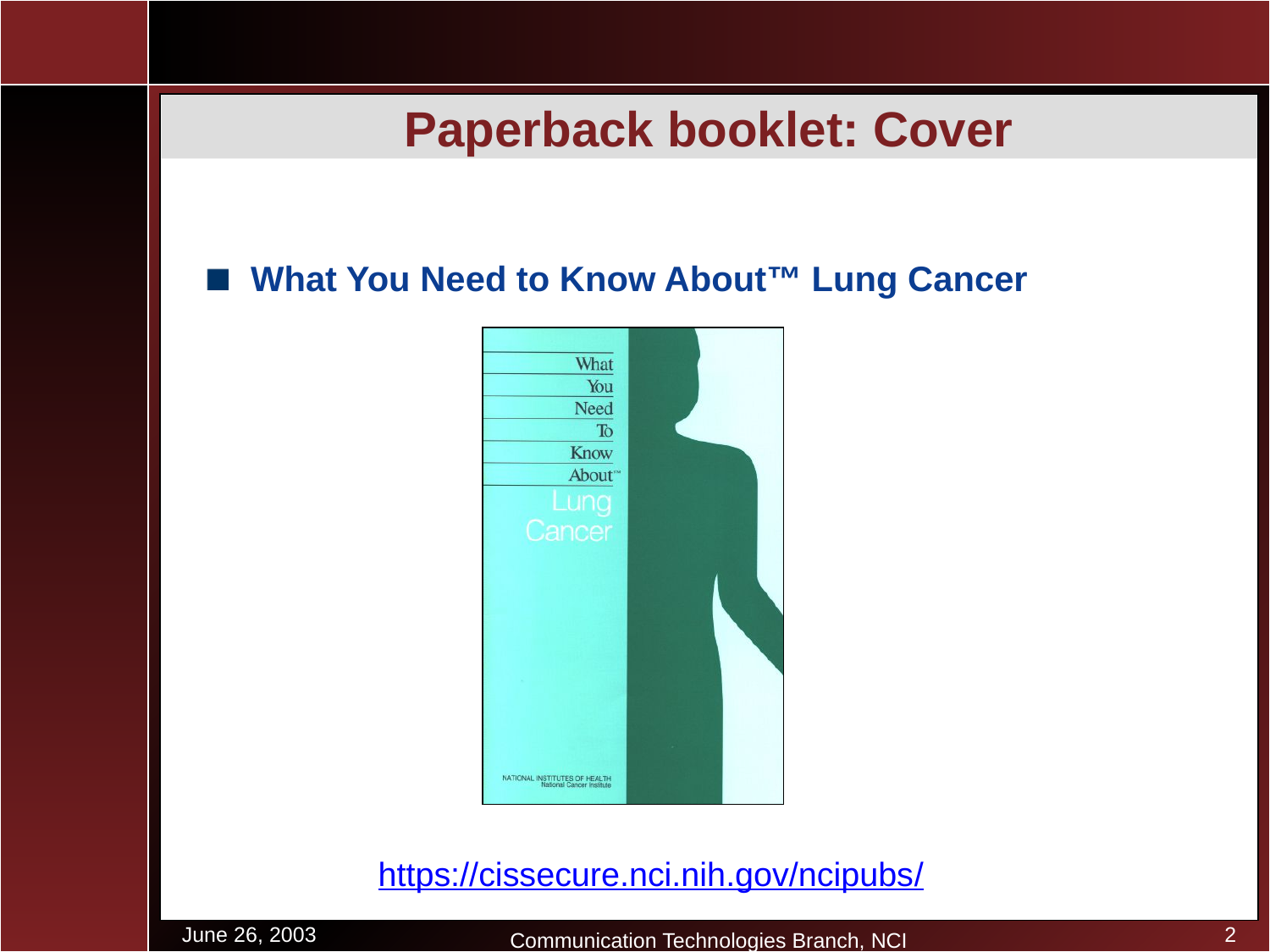

# Paperback booklet: Cover
What You Need to Know About™ Lung Cancer
https://cissecure.nci.nih.gov/ncipubs/
June 26, 2003
2
Communication Technologies Branch, NCI

## Slide 3
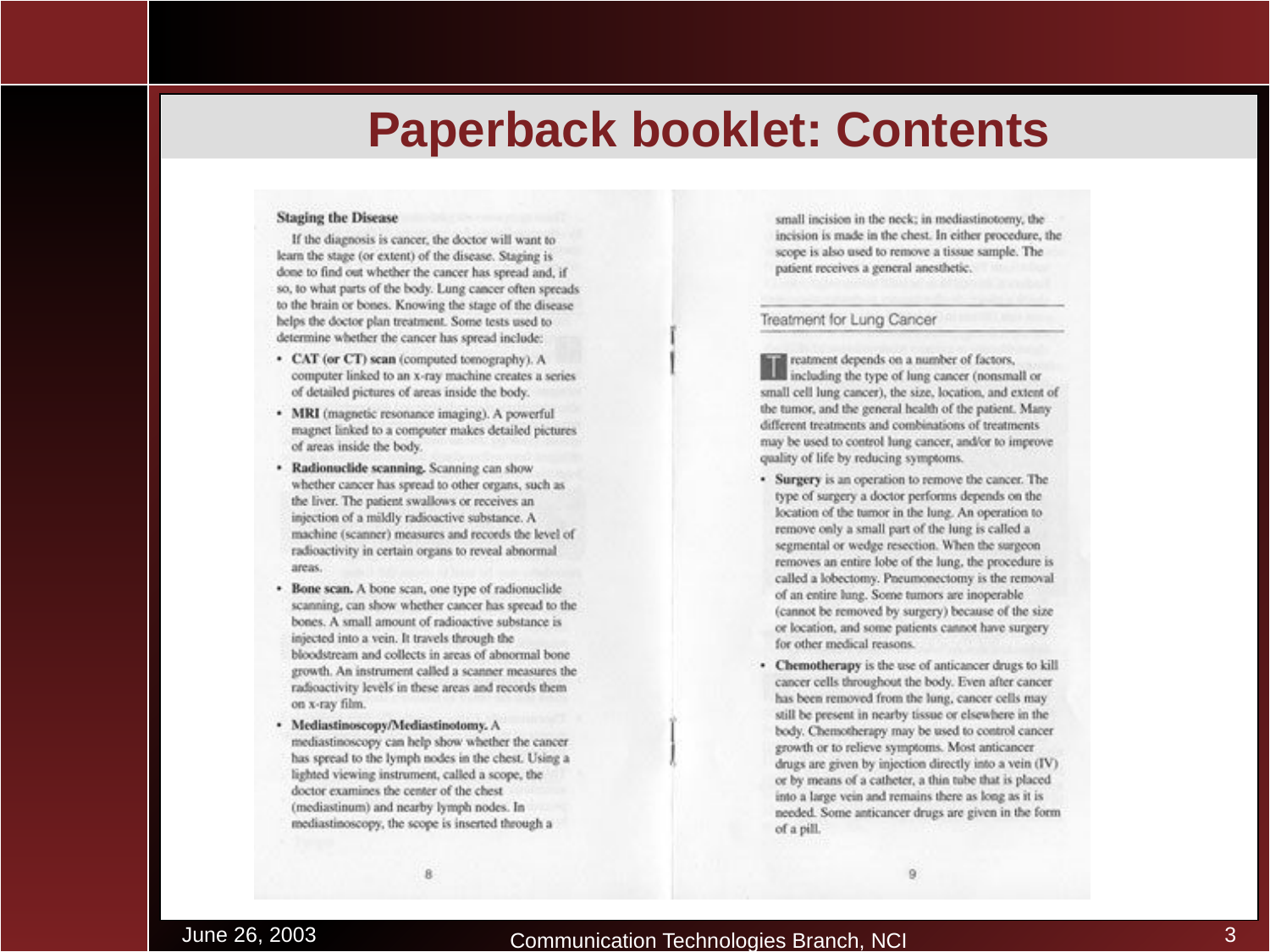

# Paperback booklet: Contents
June 26, 2003
3
Communication Technologies Branch, NCI

## Slide 4
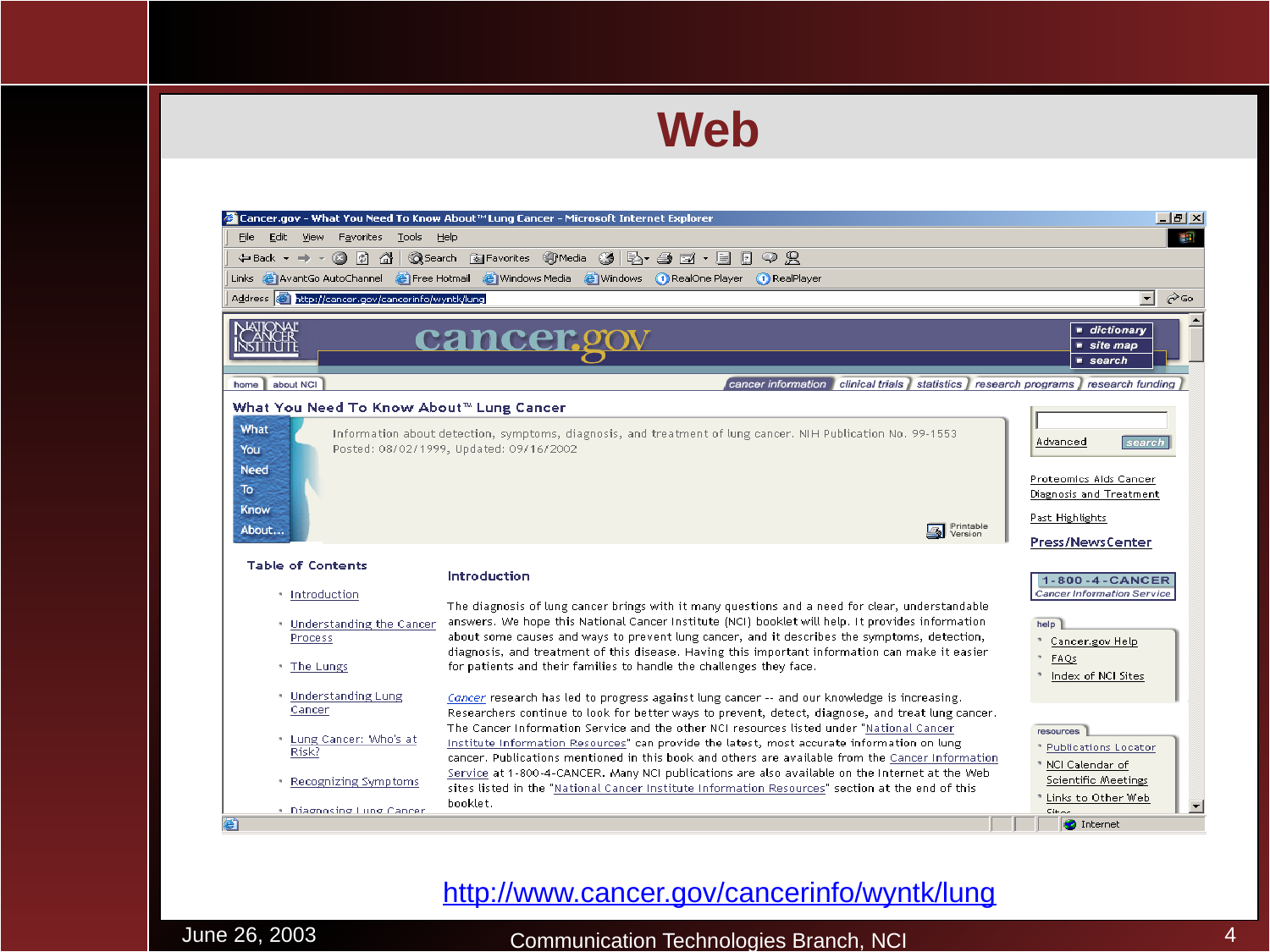

# Web
http://www.cancer.gov/cancerinfo/wyntk/lung
June 26, 2003
4
Communication Technologies Branch, NCI

## Slide 5
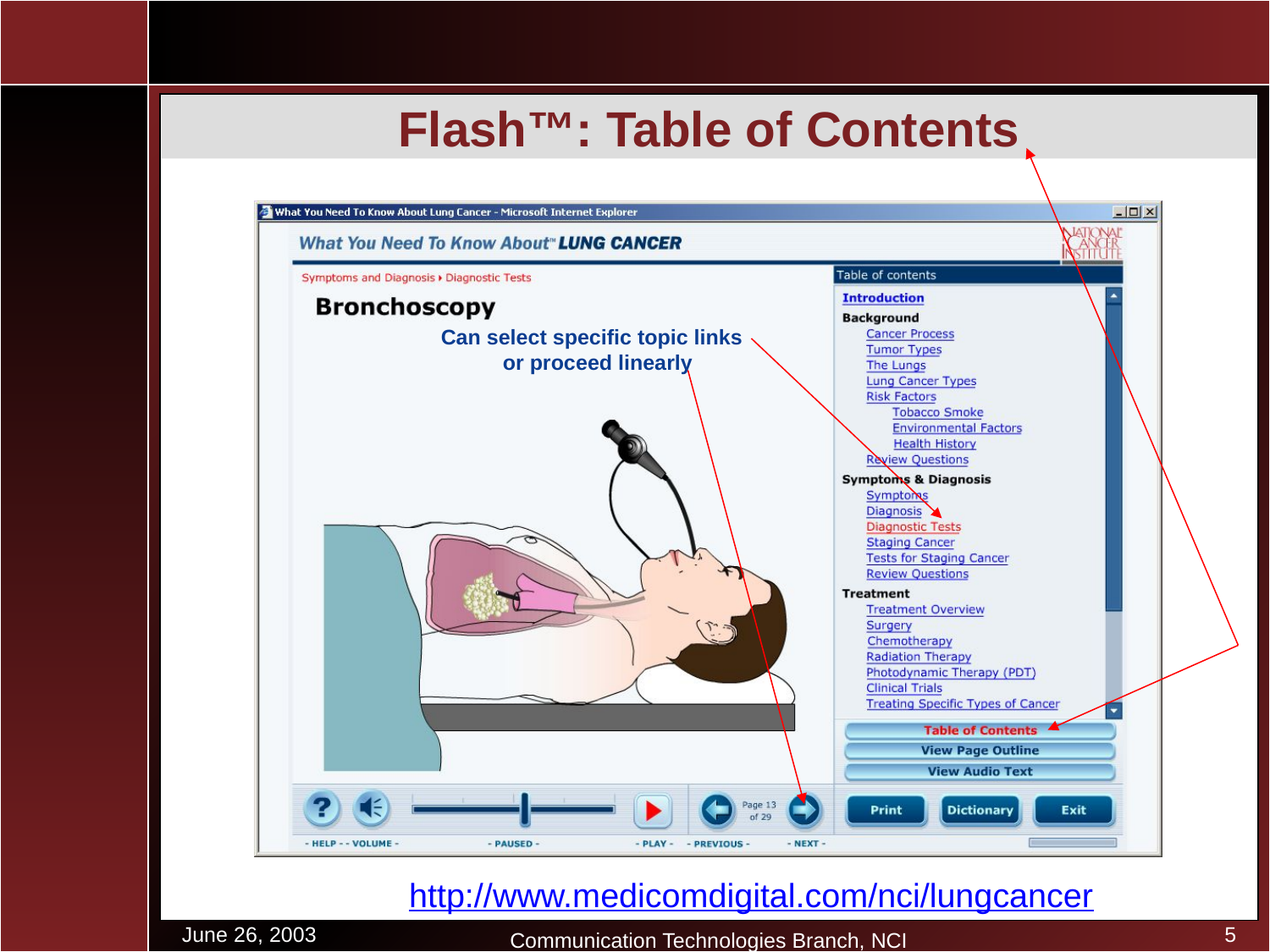

# Flash™: Table of Contents
Can select specific topic links
or proceed linearly
http://www.medicomdigital.com/nci/lungcancer
June 26, 2003
5
Communication Technologies Branch, NCI

## Slide 6
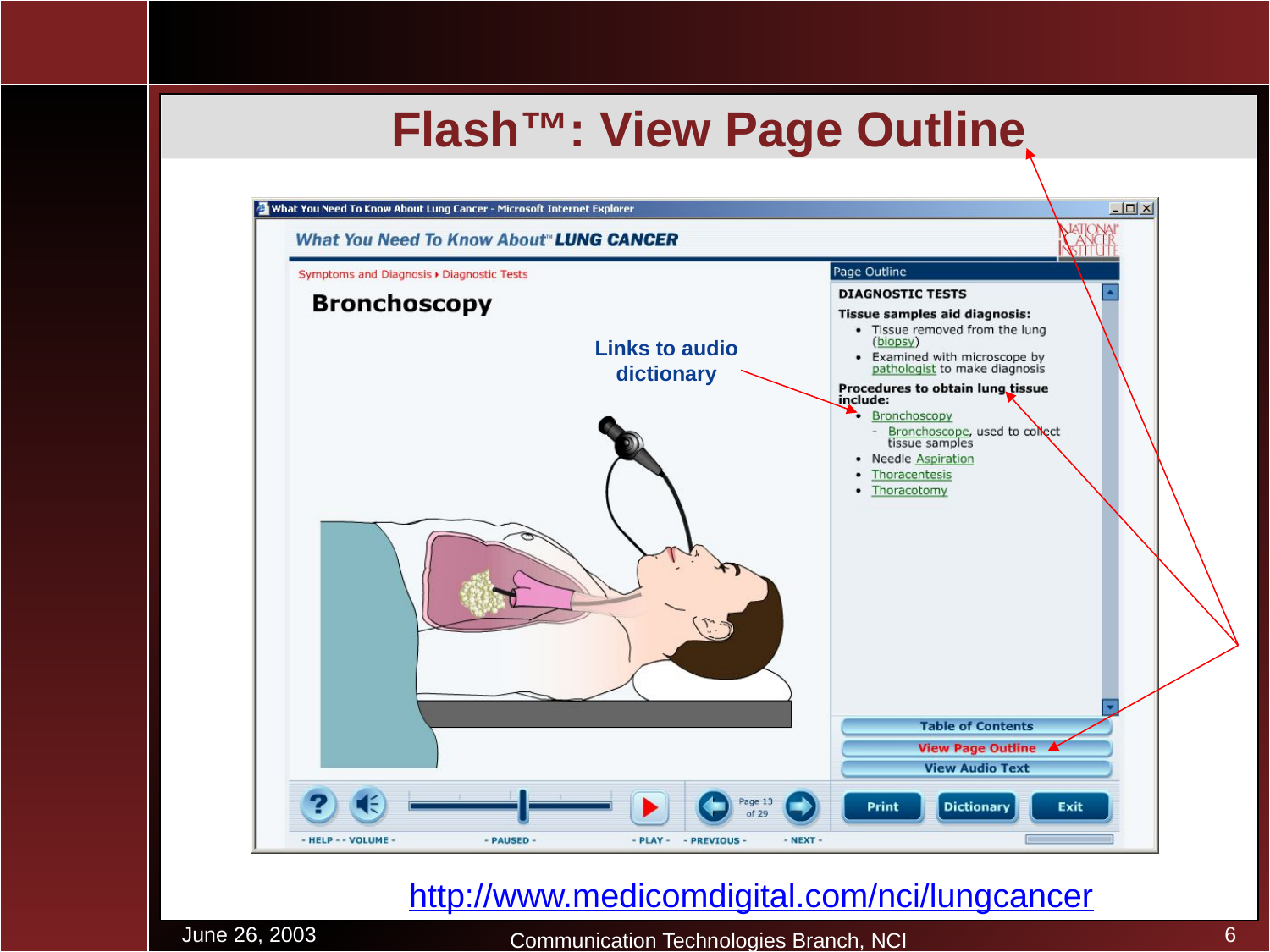

# Flash™: View Page Outline
Links to audio dictionary
http://www.medicomdigital.com/nci/lungcancer
June 26, 2003
6
Communication Technologies Branch, NCI

## Slide 7
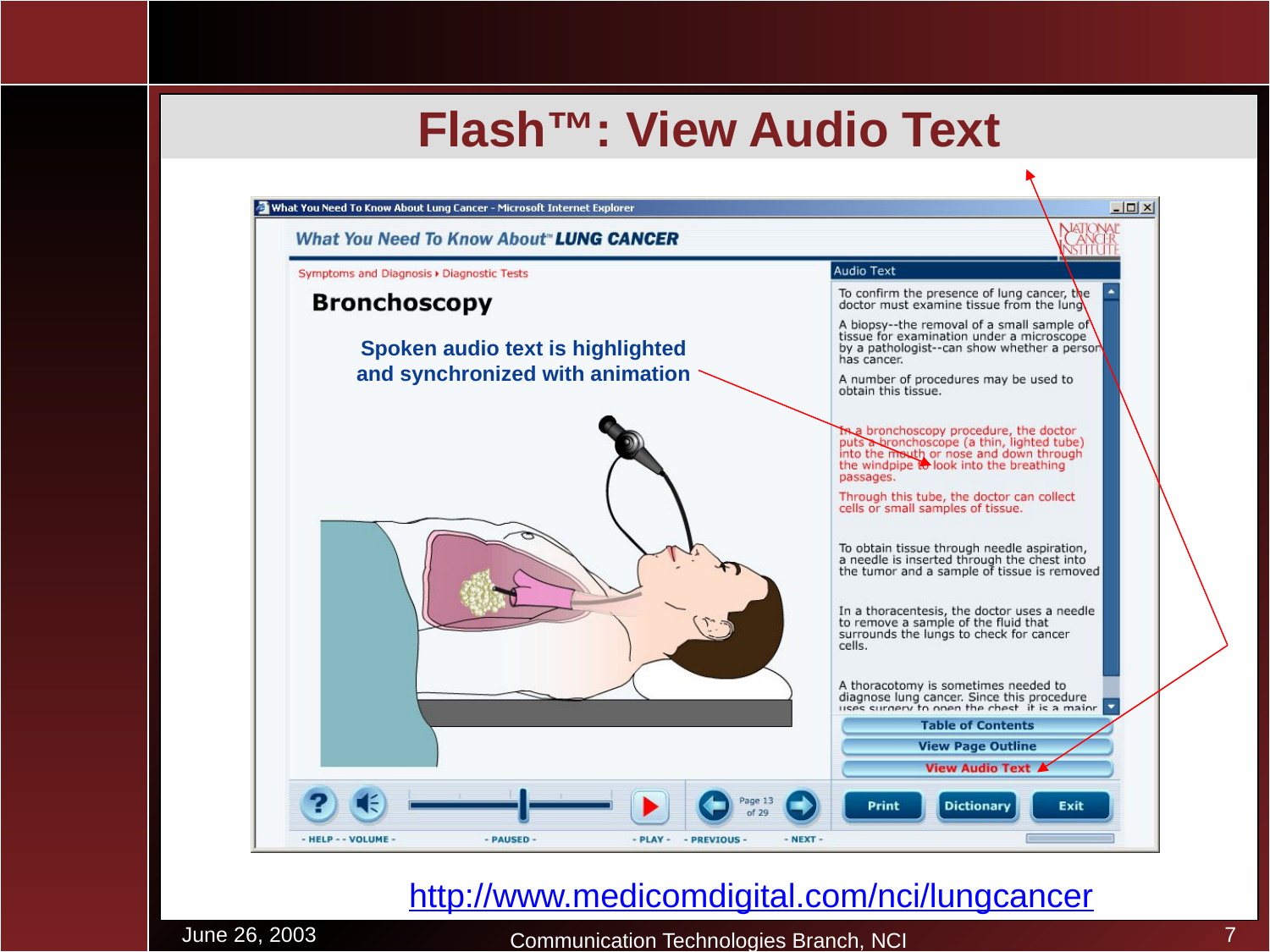

# Flash™: View Audio Text
Spoken audio text is highlighted and synchronized with animation
http://www.medicomdigital.com/nci/lungcancer
June 26, 2003
7
Communication Technologies Branch, NCI

## Slide 8
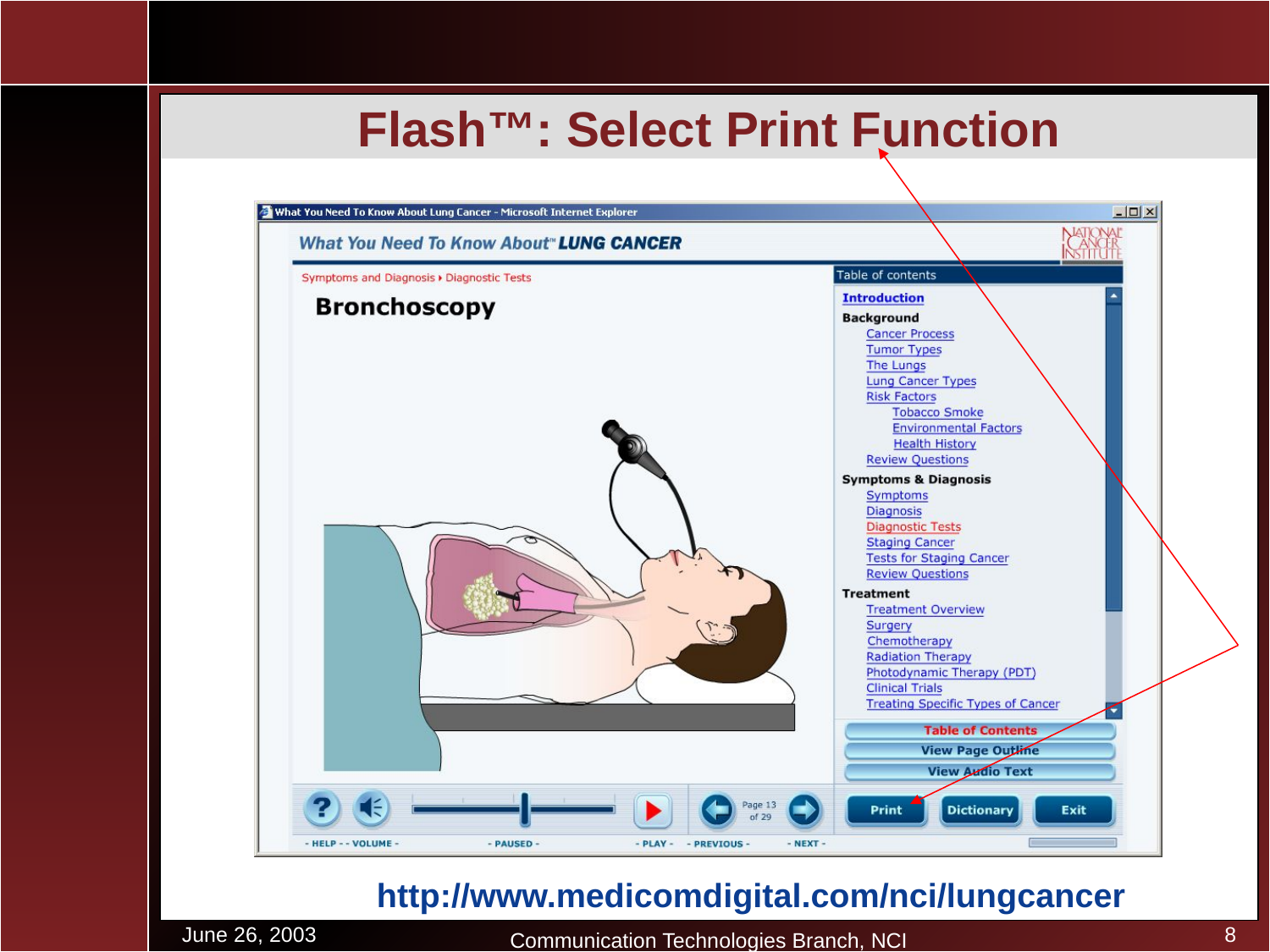

# Flash™: Select Print Function
http://www.medicomdigital.com/nci/lungcancer
June 26, 2003
8
Communication Technologies Branch, NCI

## Slide 9
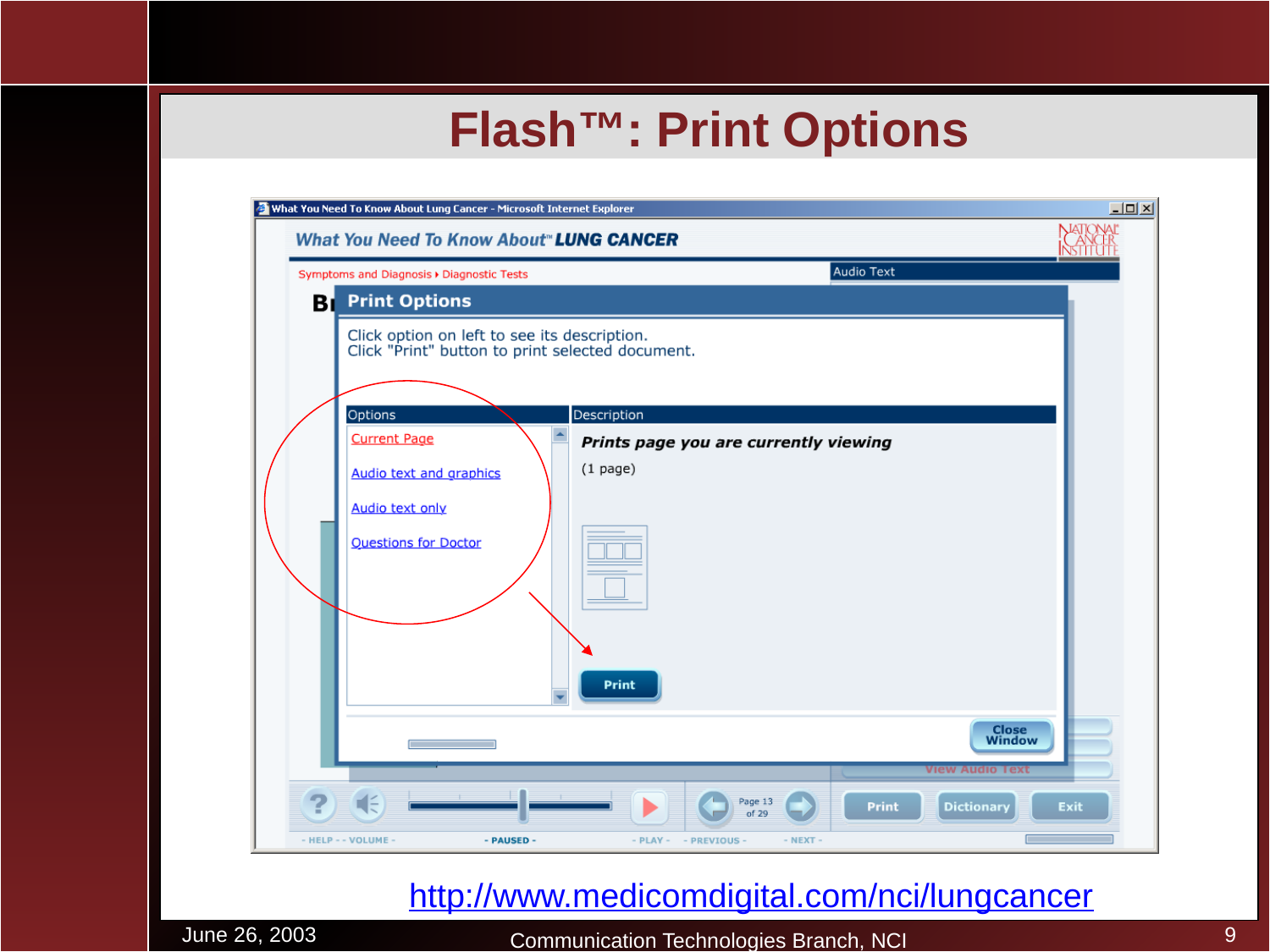

# Flash™: Print Options
http://www.medicomdigital.com/nci/lungcancer
June 26, 2003
9
Communication Technologies Branch, NCI

## Slide 10
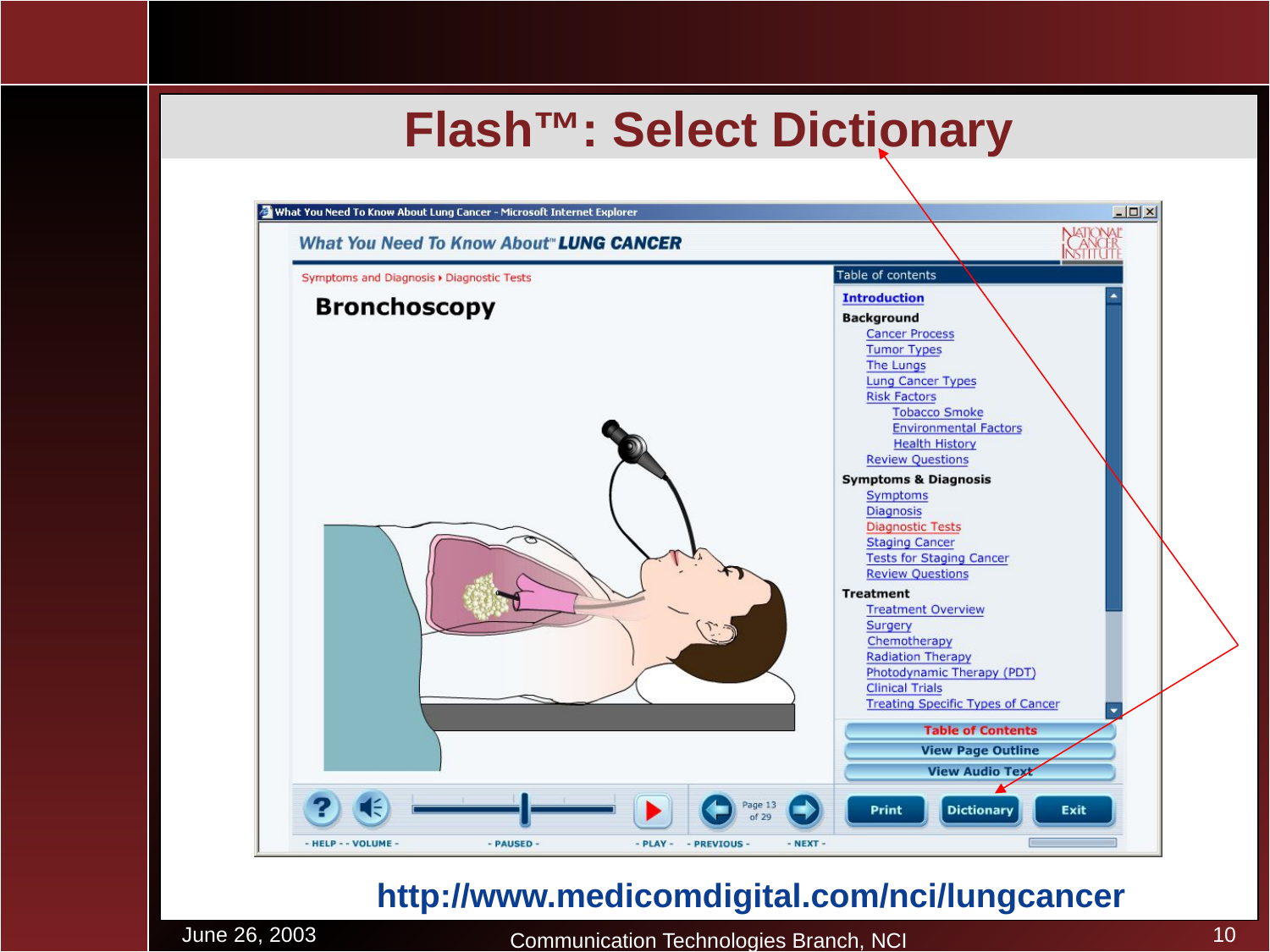

# Flash™: Select Dictionary
http://www.medicomdigital.com/nci/lungcancer
June 26, 2003
10
Communication Technologies Branch, NCI

## Slide 11
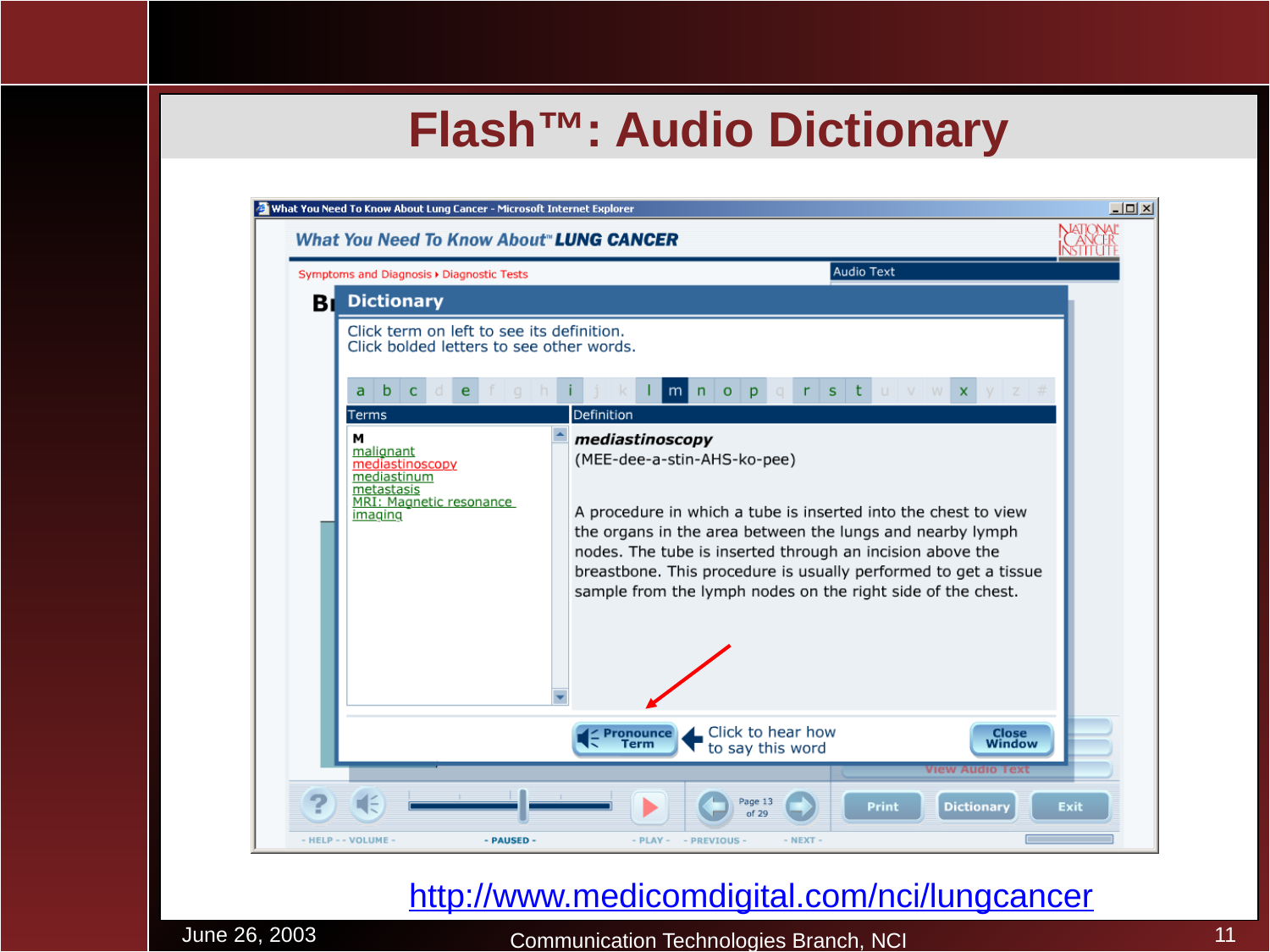

# Flash™: Audio Dictionary
http://www.medicomdigital.com/nci/lungcancer
June 26, 2003
11
Communication Technologies Branch, NCI

## Slide 12
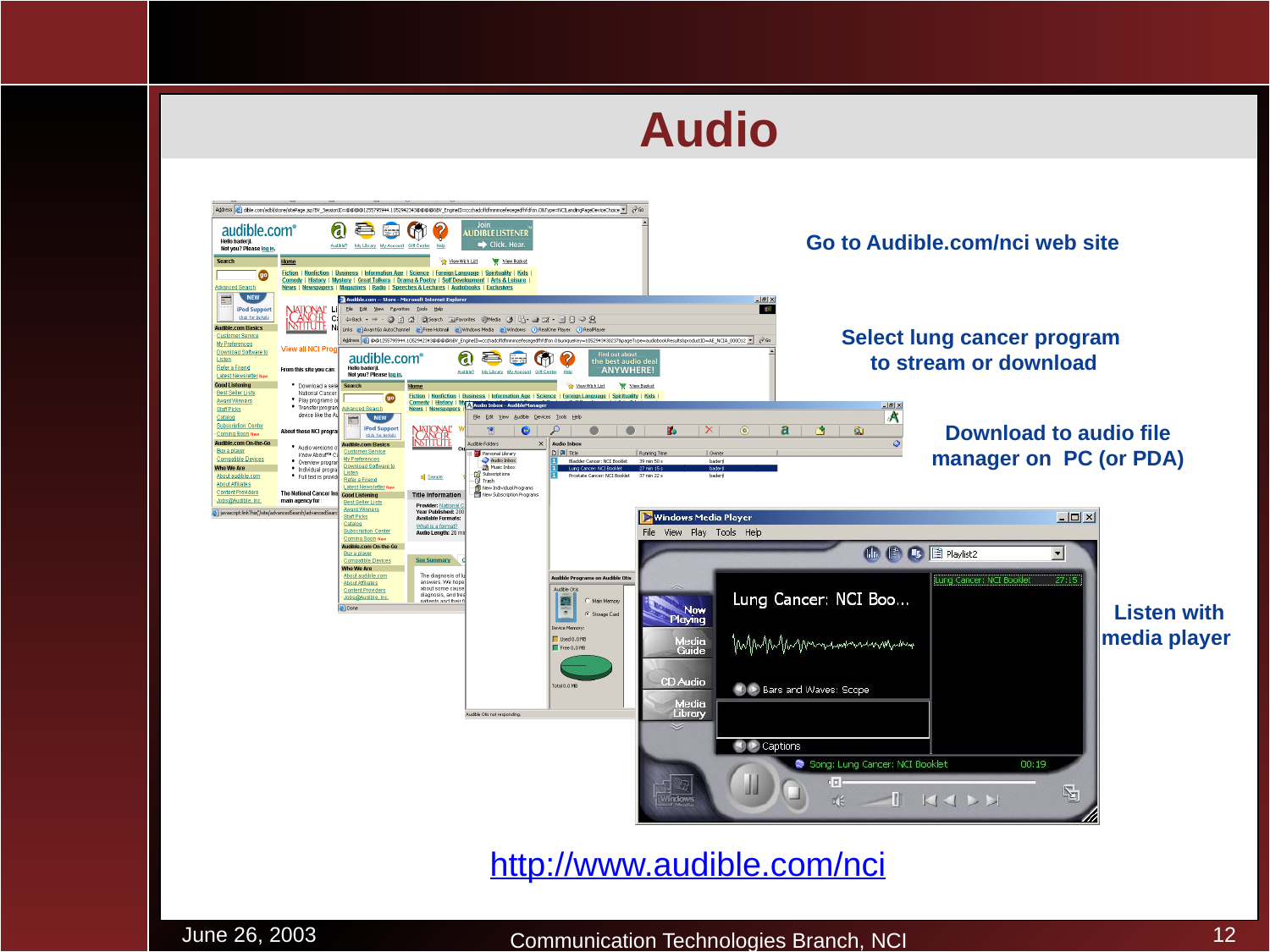

# Audio
Go to Audible.com/nci web site
Select lung cancer program
to stream or download
Download to audio file manager on PC (or PDA)
Listen with media player
http://www.audible.com/nci
June 26, 2003
12
Communication Technologies Branch, NCI

## Slide 13
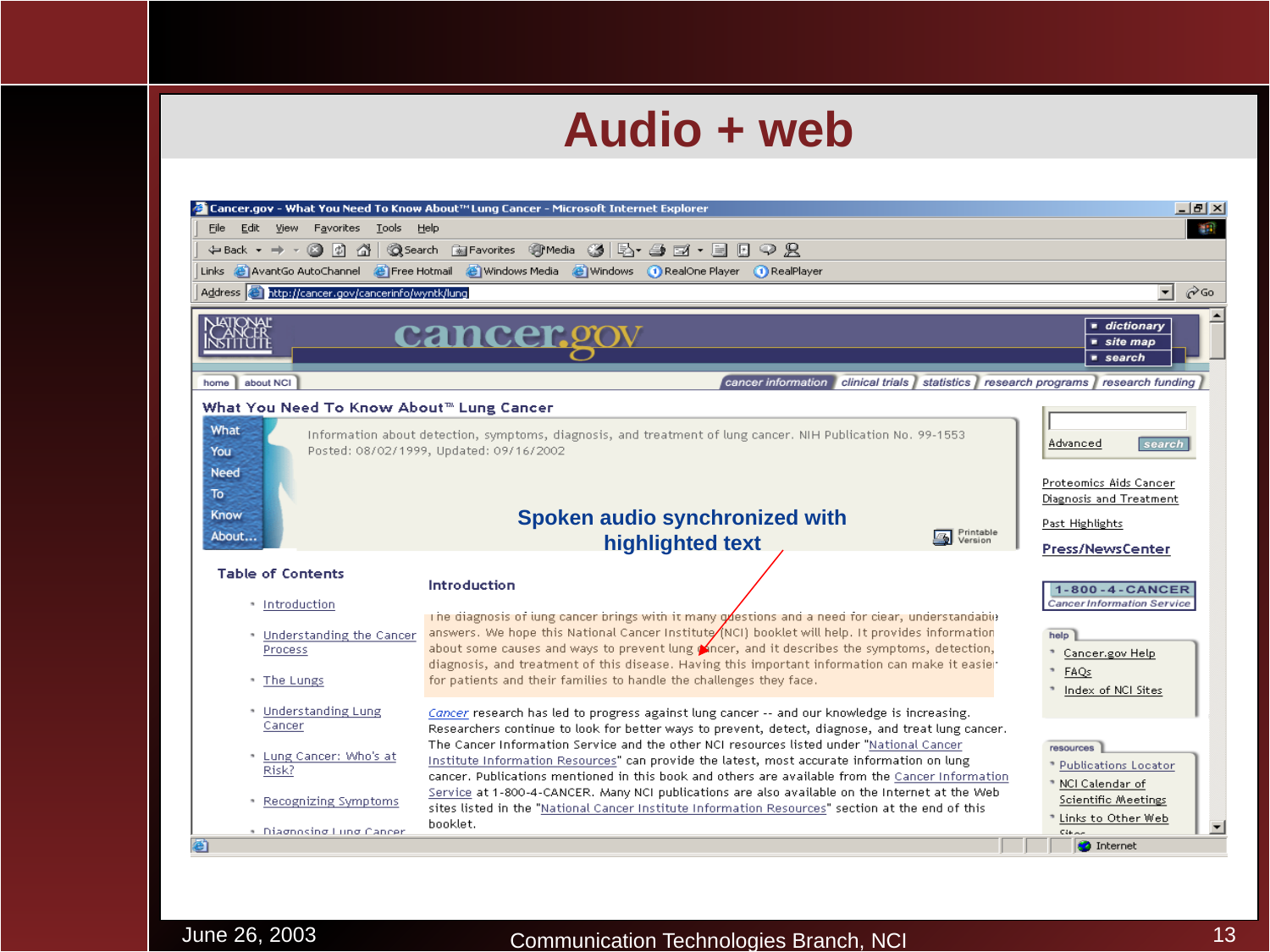

# Audio + web
Spoken audio synchronized with highlighted text
June 26, 2003
13
Communication Technologies Branch, NCI
