# Supplementary figures and images for: Evaluation of New Multimedia Formats for Cancer Communications
Source: J Med Internet Res. 2003 Aug 29;5(3):e16. doi: 10.2196/jmir.5.3.e16 (PMC1550563; doi:10.2196/jmir.5.3.e16)

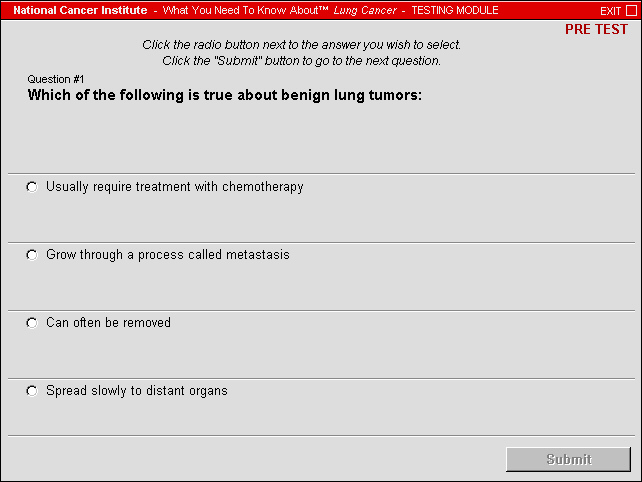

Supplement: Supplementary file 2 [file jmir_v5i3e16_app3.jpg]
